# Roles for librarians in systematic reviews: a scoping review

**DOI:** 10.5195/jmla.2018.82

**Published:** 2018-01-02

**Authors:** Angela J. Spencer, Jonathan D. Eldredge

## Abstract

**Objective:**

What roles do librarians and information professionals play in conducting systematic reviews? Librarians are increasingly called upon to be involved in systematic reviews, but no study has considered all the roles librarians can perform. This inventory of existing and emerging roles aids in defining librarians’ systematic reviews services.

**Methods:**

For this scoping review, the authors conducted controlled vocabulary and text-word searches in the PubMed; Library, Information Science & Technology Abstracts; and CINAHL databases. We separately searched for articles published in the *Journal of the European Association for Health Information and Libraries, Evidence Based Library and Information Practice,* the *Journal of the Canadian Heath Libraries Association,* and *Hypothesis.* We also text-word searched Medical Library Association annual meeting poster and paper abstracts.

**Results:**

We identified 18 different roles filled by librarians and other information professionals in conducting systematic reviews from 310 different articles, book chapters, and presented papers and posters. Some roles were well known such as searching, source selection, and teaching. Other less documented roles included planning, question formulation, and peer review. We summarize these different roles and provide an accompanying bibliography of references for in-depth descriptions of these roles.

**Conclusion:**

Librarians play central roles in systematic review teams, including roles that go beyond searching. This scoping review should encourage librarians who are fulfilling roles that are not captured here to document their roles in journal articles and poster and paper presentations.

## INTRODUCTION

Health sciences librarians have been involved with systematic reviews since this genre of publication emerged during the 1990s [1]. Since then, librarians have been most widely known for their prowess in searching for the evidence needed to create systematic reviews. Even during the early years, however, librarians and other information professionals (hereafter referred to as “librarians”) were involved in other aspects of the systematic review process [2, 3]. Two case studies during the mid-2000s suggested some potential roles for librarians in the creation of systematic reviews—such as searching, source selection, citation management, document supply, and critical appraisal. These studies were based on the limited experiences of the authors [4, 5]. Cooper’s 2013 systematic review of changing roles for health sciences librarians clearly identified participating in systematic reviews as a central role [6]. In recent years, the Medical Library Association (MLA) has regularly sponsored continuing educational training on systematic reviews.

This article offers a comprehensive inventory of roles filled by librarians in connection with conducting systematic reviews. In so doing, the authors seek to expand the potential suite of systematic review services that health sciences librarians can offer to their colleagues beyond literature searching.

## METHODS

Scoping reviews are intended to be broad, exploratory reconnaissance searches of the relevant literature to determine key characteristics of the subject [7, 8]. Our scoping review involved multiple search strategies in three databases and several websites that were used for professional communication. Our searches were conducted in February 2017, and our search strategies are described in detail in supplemental Appendix A.

We first ran a PubMed search in which we combined the Journal of the Medical Library Association OR Bulletin of the Medical Library Association OR Medical Reference Services Quarterly OR Health Information & Libraries Journal with the text-words and filters (“systematic review” OR (systematic AND review*) OR sysrev_methods[sb] OR systematic[sb]). A second PubMed search combined the Medical Subject Heading (MeSH) terms “Information Services” OR “Information Storage and Retrieval” with the text-words (librarian* OR “information scientist*” OR “information specialist*” OR informationist) and the text-words (“systematic review*” OR systematic AND review* OR sysrev_methods[sb]) OR systematic[sb]). This search was combined using the Boolean “NOT” with the first PubMed search of the four journal titles. A third PubMed search used the MeSH term “Librarians” combined with the text-words (“systematic review*” OR (systematic AND review*) OR sysrev_methods[sb] OR systematic[sb]). A fourth PubMed search used the MeSH terms “Review Literature as Topic” and “Librarians.”

We next searched the Library, Information Science & Technology Abstracts (LISTA) database for the keywords (systematic AND review*) OR “systematic review*” combined with the keywords librarian* OR “information profession*” OR “information specialist*” OR “information scientist*”. A second search in LISTA combined controlled vocabulary and text-word approaches with the descriptor “literature reviews” and keyword systemat*.

We next searched the CINAHL database for the keywords (systematic AND review*) OR “systematic review*” combined with the keywords librarian* OR “information profession*” OR “information specialist*” OR “information scientist*”. A second search of CINAHL used the descriptor “librarians” OR “health sciences librarians” AND the keyword “systematic reviews”.

Because of limited indexing coverage for CINAHL of some journals, we ran additional searches in the *Journal of the Canadian Health Libraries Association* using the term “systematic” on the main web page. We searched the *Journal of the European Association for Health Information and Libraries* using the keyword “systematic” by employing the search feature in the portable document format (PDF) viewer and scanning the table of contents. We screened the journal *Hypothesis* by searching the PDF files using the term “systematic” and scanning the tables of contents. We also scanned *Evidence Based Library & Information Practice* using the search feature on the website for the term “systematic”.

We then reviewed MLA meeting paper and poster abstracts archived on MLANET (Abstracts for MLA Annual Meeting, 2010–15 and Abstracts for MLA Annual Meeting, 2001–09; login required) for the years 2002 to 2015. We searched for the keyword “systematic” using the search feature in the PDF viewer. We also scanned the 2016 abstracts using the search feature for the meeting (MLA ’16 meeting). Finally, we searched the indexes in two textbooks on health sciences librarianship edited by M. Sandra Wood, FMLA [9, 10].

We included references that discussed any roles that librarians or information professionals performed in systematic reviews. We tried to be as broad as possible with roles that we thought a librarian might perform during a systematic review based our training, experience, and knowledge of the systematic review process. We also used two previously published articles [4, 5] to guide our identification of potential roles. We excluded references that were book reviews, advertisements for continuing education, articles on updating a systematic review, or reviews of other articles. Most of these exclusions were based on the title and abstract, but for some references we reviewed the full text for clarity.

We used Mendeley for citation management. The results of each search were placed into separate folders, with subfolders for inclusion or exclusion. We then imported all of the included abstracts into a Word document to screen for duplicates.

## RESULTS

**Figure 1:**
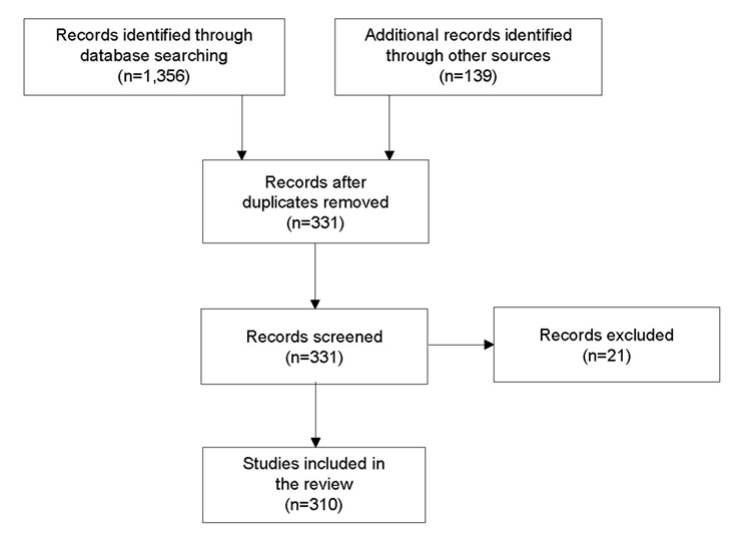
PRISMA flow diagram

Supplemental Appendix A provides the complete search methods, and Figure 1 shows the PRISMA flow diagram. Our searches resulted in 310 relevant articles, book chapters, presented papers, and posters (supplemental Appendix B). Collectively, these writings demonstrated the diverse roles that librarians play in the systematic review process. We identified a total of 18 distinct roles filled by librarians for which we provided summaries, arranged alphabetically, and highlight specific examples.

Table 1 summarizes the major professional communications channels reporting on roles of librarians in systematic reviews. The *Journal of the Medical Library Association* and *Health Information and Libraries Journal* contained the most published articles on this topic. Other peer-reviewed journals included the *Journal of the Canadian Health Libraries Association*, *Medical Reference Services Quarterly*, the *Journal of the European Association for Health Information and Libraries*, and *Evidence Based Library and Information Practice*.

**Table 1:** Major professional communications channels reporting on the roles of librarians in systematic reviews

Communications channel	Number of works
Peer-reviewed journals	
Journal of the Medical Library Association*	62
*Health Information and Libraries Journal*	53
*Journal of the Canadian Health Libraries Association*†	19
*Medical Reference Services Quarterly*	9
*Journal of the European Association for Health Information and Libraries*	9
*Evidence Based Library and Information Practice*	6
*Journal of Clinical Epidemiology*	5
*Systematic Reviews*	5
Other journal titles	22
Non-peer-reviewed journals	
*MLA News*	4
Hypothesis	2
Posters	
Posters at MLA annual meetings	54
Papers	
Papers presented MLA annual meetings	58
Book chapters	2
Total number of works	310

* Includes 6 articles from the previous title, *Bulletin of the Medical Library Association.*

† Includes 1 article from previous title, *Bibliotheca Medica Canadiana.*

Many articles and abstracts gave overviews of the roles that librarians could play in systematic reviews. For example, Harris mentioned that librarians could be involved in searching, writing the methodology, and creating a flow diagram of the article selection process [5]. She also acknowledged librarians’ knowledge of indexing and searching complexities as skills that were valued in systematic reviews. Roles mentioned in other papers or posters included planning, searching, citation management, source selection, bias assessment, data synthesis, and supplying of documents [4, 11, 12], although these roles were not always discussed in detail in the articles or abstracts.

### Citation management

Systematic reviews involve tracking a large amount of citations. These citations need to be exported into a citation management tool or spreadsheet and then documented for inclusion or exclusion. Most librarians are knowledgeable about citation management tools and often play a role in this aspect of systematic reviews. Citation management software can also be used as a screening tool [13].

### Collaboration

Collaboration determines how systematic review teams will work together and the role that each member will play in the process. As one example, an article discussed steps that librarians took after receiving multiple requests to collaborate on systematic reviews [14]. Koffel conducted a survey of authors’ reasons for why researchers chose to partner with librarians [15]. He found that working with a librarian increased the quality of searches, particularly when the librarian was knowledgeable about systematic reviews via either training or past experience. Perceived barriers included extra time for coordination with the librarian, lack of librarian subject expertise related to the systematic review topic, or cost of the service.

### De-duplication of search results

Librarians often identify and remove duplicate records from systematic review searches, which can be a time-consuming task. However, citation management tools, tools in databases, and other software can be used for de-duplicating. Some reports have examined different methods of de-duplication for specific products and databases, and analyzed the strengths and weaknesses for each method [16, 17].

### Evaluation of search strategies

Librarians review searches and past papers to evaluate the precision and sensitivity of search strategies. Librarians have retrospectively evaluated different search strategies, interfaces, and databases to demonstrate their and strengths and weaknesses [18–22].

### Formalized systematic review services

Because of the growth in demand for conducting systematic reviews, librarians are now developing formal systematic review services. Librarians at one institution described how they developed a fee-based service and educated users on what was involved in conducting a systematic review before offering further services [23].

### Impact and outcomes

Librarians advocate for their inclusion in systematic review teams by demonstrating how they improve the quality of systematic reviews. Librarians’ research has demonstrated the positive impact that they have as being part of a systematic review team, resulting in more effective search strategies [24–27].

### Indexing of database terms

Effective indexing assists in locating relevant resources. Librarians have analyzed the strengths and weaknesses of indexing for specific databases and offered suggestions on how to improve indexing [28–30].

### Peer review of search strategies

The Institute of Medicine recommends that librarians peer review search strategies for systematic reviews [31]. Sampson composed an evidence-based guideline for peer reviewing search strategies [32, 33]. Crumley documented one example of librarians peer reviewing search strategies and described the value of this process [34]. This peer-review role can sometimes overlap with the evaluation role.

### Planning

Planning is the first step for a potential team to decide if they want to conduct a systematic review. Librarians can clarify what is involved in pursuing a systematic review and how long it will take. This step provides an opportunity for librarians to discuss their potential roles in the systematic review process and set expectations. Goode described the different issues that librarians might want to discuss during the planning process [35].

### Question formulation

Librarians have extensive skills in question formulation that can be traced back to the traditional reference interview. This role of librarians in the systematic review process has rarely been mentioned [9, 10], perhaps because question formulation blends seamlessly with other librarian roles. In a randomized controlled trial, Eldredge tested the effect of library and informatics training on question formation and noted that public health professionals were able to better articulate questions after training by librarians [36].

### Reporting and documentation

Both the Institute of Medicine [31] and the Cochrane Collaboration [37] offer guidance on reporting and documenting systematic reviews. Both recommend adherence to the PRISMA statement [38]. Surprisingly, when Yoshii performed an analysis of search strategy reporting in Cochrane systematic reviews [39], none of the fifty-six papers that were reviewed contained all seven elements that the *Cochrane Handbook for Systematic Reviews of Interventions* indicated must be included*.*

### Research agenda

In 2012, the MLA Research Section published the results of the top-priority answerable research questions facing health sciences librarians [40]. These fifteen questions led to the formation of teams of librarians that conducted systematic reviews to answer these important questions [41].

### Search filters and hedges

Wilczynski stated, “A methodologic search filter is a search term or terms that select studies that are the most advanced stages of testing for clinical application” [42, 43]. Librarians have built these search filters and hedges to use in systematic reviews [44–46] and evaluated the performance of hedges and filters to see if they were accurate in their retrieval [47–78]. Librarians also developed checklists and critical appraisal tools to determine which filters might be appropriate to use for particular types of searches [49, 50].

### Searching

The Institute of Medicine requires that librarians perform searches for systematic reviews [31]. The Cochrane Collaboration also recommends having a librarian perform the search for a systematic review [37]. MLA issued a policy statement that defined librarians as expert searchers [51]. In the present scoping review, we consistently found searching to be the dominant role of librarians, as documented in the literature. Therefore, we have created subcategories to describe variations in librarians’ roles in searching for systematic reviews.

#### Databases and other resources

Several articles are devoted to searching specific databases or other resources for use in systematic reviews. The use of Scopus to help in semi-automation of manual searching, the need to search ClinicalTrials.gov, and the need to search published errata were all different approaches documented in the literature [58–60].

#### General

McGowan echoed MLA’s policy in her article, “Systematic Reviews Need Systematic Searchers,” that outlines how librarians were involved in the various steps of a systematic review. Some of the steps that she mentioned were the reference interview, development of a search strategy, source selection, and report writing. She mentioned the importance of librarians as expert searchers to construct systematic reviews [52].

#### Grey literature

In 2016, Ford moderated a session discussing different viewpoints on the use of grey literature for systematic reviews and techniques for searching grey literature [53].

#### Protocol development

The protocol for a systematic review usually states the review questions, the sources that will be searched, the inclusion and exclusion criteria, and sometimes a preliminary search of the literature. Every protocol article that we found stated that a librarian was consulted in the construction of the search query [54–57].

#### Search strategies

Search strategies typically consist of controlled vocabulary and text-word combinations that vary for each database used in the systematic review. DeLuca mentions selecting databases, selecting terms, testing the database, running the search, refining the search, and performing a manual search as steps in performing a systematic review [61].

#### Subject- or topic-specific searches

The literature documents the best search strategies for identifying adverse effects, theory, and prognosis studies [62–64].

#### Other

Some other articles on searching did not fit perfectly into a specific category, such as the use of an analytic framework when librarians grapple with searching complex questions, comparing text-word and MeSH searching, and knowing when to stop searching for abstracts [65–67]. These articles reflect the wide-ranging scope of what is involved in searching for a systematic review.

### Source selection

Librarians help guide researchers in selecting the databases and other resources that should be searched and inform team members about the strengths and weaknesses of these resources. Some of the possible resources needed for systematic reviews include databases, reference lists, personal communication, and hand searching [68]. Searching multiple databases and using a checklist is recommended for systematic reviews [69, 70]. Some non-biomedical sources need to be searched for pharmacologic policy [71]. Lam reported that the number of databases searched for systematic reviews has increased between 1994 and 2014 [72].

### Systematic reviews in librarianship

Over ninety systematic reviews on librarianship subjects have been published [73]. Some of the earliest examples include Brettle’s 2003 work on information skills training [74], Wagner’s 2004 study on measuring the effectiveness of clinical medical librarian programs [75], and Weightman’s 2005 evaluation of the impact of library services on patient care [76]. Teams around the world are currently performing additional systematic reviews on high-priority librarianship topics [77, 78].

### Teaching

Librarians play a role in teaching others, including other librarians, about how to perform systematic reviews. Harris identified this teaching role in one of the first communications on librarians’ roles in conducting systematic reviews [79]. Traditional classroom, flipped classroom, and virtual mentoring have all been used to teach librarians or researchers how to perform systematic reviews [80–83]. These methods reflect the diversity of learner needs and approaches that librarians use to teach others about systematic reviews.

### Technological and analytical tools

Librarians are developing and using technological tools to aid in systematic review production. McKibbon employed the use of the Capture Mark-Recapture method to estimate how many studies might be found for a specific systematic review [84]. Bradford’s Law of Scattering has provided another analytical tool for projecting the number of references for a systematic review [85].

### Other roles

Not all the reviewed works fit into the above categories. For instance, Sampson promoted submitting systematic reviews to the *Journal of the Medical Library Association* in 2014 [86], and de Jonge encouraged librarians to share literature search blocks among themselves [87]. Search blocks are saved strategies that have not been verified and are often shared with other librarians in the same institution. Gore provided an overview for managers who are not familiar with systematic reviews on how librarians are involved with systematic reviews and the areas where they might need support such as training [88]. Bullers analyzed how much time librarians spent on systematic reviews [89]. Also, Foster created an MLA special interest group to share knowledge about systematic reviews [90].

## DISCUSSION

This scoping review produced a bibliography of 310 works related to librarians’ 18 core roles in systematic reviews. Our scoping review uncovered both expected and less expected roles that librarians performed in systematic reviews. Expected roles included searching, source selection, and evaluation, whereas less documented roles were planning, question formulation, and peer review. It is important to note that some of the roles that Beverley and Harris mentioned (e.g., data abstraction, data extraction, bias assessment, critical appraisal, data synthesis, document supply, report writing) [4, 5] were not further described by other works, and therefore, we did not include these among our list of 18 roles.

We encourage those who provide these services to communicate about their experiences. The diversity of these roles demonstrates many of the roles that librarians can play in the systematic review process. This inventory should serve as a helpful checklist for librarians to showcase the roles that they can play while still in the planning stages of systematic reviews. While many librarians might be familiar with many of these specific roles, some librarians will be not be familiar with all of them. For example, many hospital librarians might not provide a comprehensive suite of services for systematic reviews due to the time commitment involved but might be able to use this inventory to see what specific roles they can provide to support their researchers.

We reasoned at the outset of this research project that we should capture the grey literature related to librarians’ roles in systematic reviews to provide a more complete spectrum of roles. Very little of this grey literature resulted in publication in peer-reviewed journals. Thus, the presence of many meeting papers and posters reinforced the frequent observation that one needs to consult the grey literature when accessing a professional knowledgebase.

We likely missed a few references for articles, papers, or posters in cases where librarians were not acknowledged, their roles were not documented in professional communications, or their job titles might have eluded the search strategies’ reach. We acknowledge that some of the roles overlap and that many of the articles, papers, and posters included more than one role. Also, some references might not have been indexed using the terms searched.

Our scoping review indicates that librarians play central roles in systematic review teams, including roles beyond searching. We hope that this scoping review broadens librarians’ knowledge of the roles that they currently play and that librarians can use our findings as a tool to educate others on the diverse roles that they can offer.

## Supplemental Files

Appendix A Database search strategies

Appendix B Included papers bibliography
