# Supplementary material for: Roles for librarians in systematic reviews: a scoping review
Source: J Med Libr Assoc. 2018 Jan 2;106(1):46–56. doi: 10.5195/jmla.2018.82 (PMC5764593; doi:10.5195/jmla.2018.82)
Supplement: Appendix A [file jmla-106-46-s001.pdf]

Journal of the Medical Library Association

Supplemental content to
J Med Libr Assoc. Jan;106(1):dx.doi.org/10.5195/jmla.2018.82
www.jmla.mlanet.org
© Spencer, Eldredge, 2018

Roles for librarians in systematic reviews: a scoping review

Angela J. Spencer, MLS; Jonathan D. Eldredge, MLS, PhD, AHIP

APPENDIX A

Database search strategies

Database	Search	Found	Viable	After de-duplication
PubMed, 1st search	((((((("Journal of the Medical Library Association : JMLA"[Jour]) OR "Bulletin of the Medical Library Association"[Jour]) OR "Medical reference services quarterly"[Jour]) OR ("Health information and libraries journal"[Jour]))) AND ((((systematic review) OR (systematic AND review*)) OR sysrev_methods[sb]) OR systematic[sb])))	530	122	0
PubMed, 2nd search	(((((((information services[mesh]) OR (information storage and retrieval[mesh])))) AND (((("systematic review*") OR ((systematic AND review*))) OR sysrev_methods[SB]) OR systematic[sb])) AND ((((informationist) OR "information specialist*") OR "information scientist*") OR librarian*))) NOT ((("Bull Med Libr Assoc"[Journal] OR "J Med Libr Assoc"[Journal] OR "Med Ref Serv Q"[Journal] OR "Health Info Libr J"[Journal])))	85	18	15
PubMed, 3rd search	((("systematic review*" OR (systematic AND review*) OR sysrev_methods[SB] OR systematic[sb]))) AND librarians[mesh]	129	25	1
PubMed, 4th search	review literature as topic[mesh] AND librarian [mesh]	14	6	1
Library, Information Science & Technology Abstracts (LISTA), 1st search	("systematic review" OR (systematic AND review*)) AND (librarian* OR "information profession*" OR "information scientist*" OR "information specialist*")	282	57	14
LISTA, 2nd search	DE "literature reviews" and systemat*	80	4	1
CINAHL, 1st search	("systematic review*" OR (systematic AND review*)) AND (librarian* OR "information profession*" OR "information scientist*" OR "information specialist*")	186	50	18
CINAHL, 2nd search	MH "systematic review" AND (MH "librarians" OR MH "Health sciences Librarians"	50	25	0
