# Supplementary material for: Roles for librarians in systematic reviews: a scoping review
Source: J Med Libr Assoc. 2018 Jan 2;106(1):46–56. doi: 10.5195/jmla.2018.82 (PMC5764593; doi:10.5195/jmla.2018.82)
Supplement: Appendix B [file jmla-106-46-s002.pdf]

JMLA Journal of the Medical Library Association

SC

Supplemental content to
J Med Libr Assoc. Jan;106(1):dx.doi.org/10.5195/jmla.2018.82
www.jmla.mlanet.org
© Spencer, Eldredge, 2018

Roles for librarians in systematic reviews: a scoping review

Angela J. Spencer, MLS; Jonathan D. Eldredge, MLS, PhD, AHIP

APPENDIX B

Included papers bibliography

Overview of roles

Ascher MT, Foster MJ, MacEachern M, Townsend WA. Beyond the search: expanding role of the librarian in the systematic review process. Presented at MLA '16, the 116th Annual Meeting of the Medical Library Association; Toronto, ON, Canada; May 13–18, 2016.

Balduini A, Guizzetti,G, Molinari S, Truccolo I, Motta S, Bernardini F, Curti M. [The role of the scientific librarian in HTA: what is the status quo and what are the prospects]. Recenti progressi in medicina. 2013 Oct;104(10):542–44.

Beverley CA, Booth A, Bath PA. The role of the information specialist in the systematic review process: a health information case study. Health Inf Libr J. 2003 Jun;20(2):65–74.

Cunningham DJ. A health sciences librarian's role as a member of a systematic review team. MLA News. 2011 Feb;51(2):9–10.

Dudden RF, Protzko SL. The systematic review team: contributions of the health sciences librarian. Med Ref Serv Q. 2011;30(3):301–15.

Foster MJ. An overview of the role of librarians in systematic reviews: from expert searcher to project manager. J Eur Assoc Health Inf Libr. 2015;11(3):3–7.

Greenley SL. Beyond searching: practical advice for increasing your role in systematic reviews. Presented at MLA '11, the 111th Annual Meeting of the Medical Library Association; Minneapolis, MN; May 13–18, 2011.

Harris MR. The librarian's roles in the systematic review process: a case study. J Med Libr Assoc. 2005 Jan;93(1):81–7.

Justice EM, Belleh E, Easterby-Gannett S, Moran D, Evans J, Risenberg LA. Finding our way in the world of systematic reviews: hospital librarians contribute to the creation of systematic reviews. Poster presented at MLA '13, the 113th Annual Meeting of the Medical Library Association; Boston, MA; May 3–8, 2013.

Lightfoot D, Epworth A, Friesen F, Lan CLT, Lolova E, Morant B. Understanding and enhancing library services in support of systematic reviews. J Can Health Libr Assoc. 2015 Aug;36(2):88.

Mann MK, Huntley A, Purdy S, Huws DW, Paranjothy S, Brindle P, Thomas R, Elwyn G. The role of the librarian in conducting systematic reviews. Presented at MLA '13, the 113th Annual Meeting of the Medical Library Association; Boston, MA; May 3–8, 2013.

Mann MK, Huntley A, Purdy S. Steps to conducting a systematic review. Poster presented at MLA '13, the 113th Annual Meeting of the Medical Library Association; Boston, MA; May 3–8, 2013.

McKibbon KA. Systematic reviews and librarians. Libr Trends. 2006 Summer;55(1):202–15.

Mead TL, Richards DT. Librarian participation in meta-analysis projects. Bull Med Libr Assoc. 1995 Oct;83(4):461–4.

Price C, Riese VG, Lobner K, Blanck JF, Anton B. Beyond the search: librarian involvement on the systematic review team. Presented at MLA ′15, the 115th Annual Meeting of the Medical Library Association; Austin, TX; May 15–20, 2015.

Romero L. Librarian as co-author, co-reviewer and consultant: systematic review search service and advanced search skills consultant. Health Inform. 2015;24(2):13–25.

Ross-White A. Librarian involvement in systematic reviews at Queen's University: an environmental scan. J Can Health Libr Assoc. 2016;37(2):39–43.

Sathe NA. Librarian contributions to the systematic review process. MLA News. 2011 Jun/Jul;51(6):14–5.

McGowan JL, Sampson M, Lefebvre C, Salzwedel D. To the editor: response to Nila A. Sathe's "Librarian contributions to the systematic review process." MLA News. 2011 Aug;51(7):10.

Schell CL, Rathe RJ. Meta-analysis: a tool for medical and scientific discoveries. Bull Med Libr Assoc. 1992 Jul;80(3)219–22.

Shell L, Hofstetter S, Carlock D, Amani J. Survivor's guide for the novice: a simplified model for a collaborative systematic review. J Hosp Librariansh. 2006;6(4):1–12.

Stenbaek DE, Jensen MF. The information specialist and health technology assessment challenges and opportunities. J Eur Assoc Health Inf Libr. 2006;2(2):37–9.

Townsend W, MacEachern MP, Zeylikovich I. A study of librarian involvement in locally created systematic reviews. Poster presented at MLA ′13, the 113th Annual Meeting of the Medical Library Association; Boston, MA; May 3–8, 2013.

Vrabel M. Assisting the oncology advanced practice nurse (APN) in fostering evidence-based practice: the role of the librarian. Poster presented at MLA ′03, the 103rd Annual Meeting of the Medical Library Association; San Diego, CA; May 2–7, 2003.

Werner DA. All but the search: supporting systematic reviews without performing the literature search. Poster presented at MLA ′13, the 113th Annual Meeting of the Medical Library Association; Boston, MA; May 3–8, 2013.

Citation management

Bramer WM, Milic J, Mast F. Reviewing retrieved references for inclusion in systematic reviews using EndNote. J Med Libr Assoc. 2017 Jan;105(1):84–7. DOI: http://dx.doi.org/10.5195/jmla.2017.111.

Brennan D. Simple export of journal citation data to Excel using any reference manager. J Med Libr Assoc. 2016 Jan;104(1):72–5. DOI: http://dx.doi.org/10.3163/1536-5050.104.1.012.

Gomis M, Gall C, Brahmi FA. Web-based citation management compared to EndNote: options for medical sciences. Med Ref Serv Q. 2008 Fall;27(3):260–71.

Peters MD. Managing and coding references for systematic reviews and scoping reviews in EndNote. Med Ref Serv Q. 2017 Jan–Mar;36(1):19–31.

Collaboration

Berendsen M, Hitchcock K. Connecting with faculty to define the role of librarians in the systematic review process. Poster presented at MLA ′10, the 110th Annual Meeting of the Medical Library Association; Washington, DC; May 21–26, 2010.

Supplemental content to
J Med Libr Assoc. Jan;106(1):dx.doi.org/10.5195/jmla.2018.82
www.jmla.mlanet.org
© Spencer, Eldredge, 2018

Campbell S, Dorgan M. What to do when everyone wants you to collaborate: managing the demand for library support in systematic review searching. J Can Health Libr Assoc. 2015;36(1):11–9.

Hofstetter S, Shell LB, Carlock, DM, Amani J. Support for the systematic review in evidence-based practice: a simplified model for a multi-campus research team. Poster presented at MLA ’06, the 106th Annual Meeting of the Medical Library Association; Phoenix, AZ; May 19–24, 2006.

Jerome R, Walden R, McKoy JN, McPheeters M, Hartmann K, Giuse NB. Leveraging librarians’ skills in searching and critical appraisal in a systematic review collaboration. Presented at MLA ’11, the 111th Annual Meeting of the Medical Library Association; Minneapolis, MN; May 13–18, 2011.

Koffel J. Survey of systematic review authors to determine rates of librarian involvement benefits, roles, and barriers to collaboration. Presented at MLA ’15, the 115th Annual Meeting of the Medical Library Association; Austin, TX; May 15–20, 2015.

Kuntz G, Seymour AK, Umscheid CA, Williams K. Supporting evidence-based medicine in an academic health system: a unique partnership between the center for evidence-based practice and the biomedical library. Poster presented at MLA ’07, the 107th Annual Meeting of the Medical Library Association; Philadelphia, PA; May 18–23, 2007.

Mann MK, Weightman A. Going beyond the traditional roles: collaboration the key to survival. Poster presented at MLA ’13, the 113th Annual Meeting of the Medical Library Association; Boston, MA; May 3–8, 2013.

Martin JR, Kramer SS, Chisholm-Burns MA, Lee JK, Spivey CA, Slack MK, Herrier RN. Librarians as members of an interdisciplinary team conducting a systematic review of pharmacists’ impact on direct patient care. Presented at MLA ’11, the 111th Annual Meeting of the Medical Library Association; Minneapolis, MN; May 13–18, 2011.

Mi M, Li J, Wu L, Wu W, Zhang Y. Reflective practice for professional development through a collaborative systematic review project. Poster presented at MLA ’16, the 116th Annual Meeting of the Medical Library Association; Toronto, ON, Canada; May 13–18, 2016.

Swinkels A, Briddon J, Hall J. Two physiotherapists, one librarian and a systematic literature review: collaboration in action. Health Inf Libr J. 2006 Dec;3(4):248–56.

Whalen KJ. Lead, follow, or get out of the way: teaming up with nursing faculty to research, write, and publish systematic reviews. Presented at MLA ’11, the 111th Annual Meeting of the Medical Library Association; Minneapolis, MN; May 13–18, 2011.

De-duplication of search results

Bramer WM, Giustini D, de Jonge GB, Holland L, Bekhuis T. De-duplication of database search results for systematic reviews in EndNote. J Med Libr Assoc. 2016 Jul;104(3):240–3. DOI: http://dx.doi.org/10.3163/1536-5050.104.3.014. Correction in: J Med Libr Assoc. 2017 Jan;105(1):111. DOI: http://dx.doi.org/10.5195/jmla.2017.128.

Kwon Y, Lemieux M, McTavish J, Wathen N. Identifying and removing duplicate records resultant from systematic review searches: a comparative investigation. J Can Health Libr Assoc. 2015;36(2):83–4.

Kwon Y, Lemieux M, McTavish J, Wathen N. Identifying and removing duplicate records from systematic review searches. J Med Libr Assoc. 2015 Oct;103(4):184–8. DOI: http://dx.doi.org/10.3163/1536-5050.103.4.004.

JMLA Journal of the Medical Library Association

Supplemental content to
J Med Libr Assoc. Jan;106(1):dx.doi.org/10.5195/jmla.2018.82
www.jmla.mlanet.org
© Spencer, Eldredge, 2018

<u>**Evaluation of search strategies**</u>

Aalai E, Gleghorn C, Webb A, Glover SW. Accessing public health information: a preliminary comparison of CABI's GLOBAL HEALTH database and MEDLINE. Health Inf Libr J 2009 Mar;26(1):56–62.

Bramer WM, Giustini D, Kramer BM, Anderson P. The comparative recall of Google Scholar versus PubMed in identical searches for biomedical systematic reviews: a review of searches used in systematic reviews. Syst Rev. 2013 Dec 23;2:115.

Bramer WM, Giustini D, Kramer BM. Comparing the coverage, recall, and precision of searches for 120 systematic reviews in EMBASE, MEDLINE, and Google Scholar: a prospective study. Syst Rev. 2016 Mar 1;5:39.

Bramer WM. Comparing the effectiveness of conceptual search methods: is a fast approach sufficient for the production of sound systematic reviews: a prospective, double-blinded, controlled study. Poster presented at MLA '16, the 116th Annual Meeting of the Medical Library Association; Toronto, ON, Canada; May 13–18, 2016.

Bramer WM. Variation in the number of hits for complex searches in Google Scholar. J Med Libr Assoc. 2016 Apr;104(2):143–5. DOI: http://dx.doi.org/10.3163/1536-5050.104.2.009.

Craven J, Jefferies J, Kendrick J, Nicholls D, Boynton J, Frankish R. A comparison of searching the Cochrane library databases via CRD, Ovid and Wiley: implications for systematic searching and information services. Health Inf Libr J. 2014 Mar;31(1):54–63.

Eisinga A, Siegfried N, Clarke M. The sensitivity and precision of search terms in phases I, II and III of the Cochrane highly sensitive search strategy for identifying reports of randomized trials in MEDLINE in a specific area of health care-HIV/AIDS prevention and treatment intervention. Health Inf Libr J. 2007 Jun;24(2):103–9.

Golder S, Loke YK, Zorzela L. Comparison of search strategies in systematic reviews of adverse effects to other systematic reviews. Health Inf Libr J. 2014 Jun;31(2):92–105.

Golder S, Loke YK. Failure or success of electronic search strategies to identify adverse effects data. J Med Libr Assoc. 2012 Apr;100(2):130–4. DOI: http://dx.doi.org/10.3163/1536-5050.100.2.012.

Greenley SL. Building on existing evidence: comparing MEDLINE systematic review search strategies for everyday use. Poster presented at MLA '08, the 108th Annual Meeting of the Medical Library Association; Chicago, IL; May 16–21, 2008.

Koffel J, Rethlefsen ML. Reproducibility of systematic review search strategies in cardiology, surgery, and pediatrics journals. Presented at MLA '14, the 114th Annual Meeting of the Medical Library Association; Chicago, IL; May 16–21, 2014.

Lackey M, Lee JGL, Ylioja T. How systematic are they? outing high-quality lesbian, gay, bisexual, transgender (LGBT) health systematic reviews and meta-analyses. Poster presented at MLA '16, the 116th Annual Meeting of the Medical Library Association; Toronto, ON, Canada; May 13–18, 2016.

Landry T, Amar-Zifkin A. Quantifying the systematic search: an analysis of retrieval in published Cochrane systematic reviews. Presented at MLA '16, the 116th Annual Meeting of the Medical Library Association; Toronto, ON, Canada; May 13–18, 2016.

McGraw M. Analysis of published papers supported by Cleveland Health Sciences Library systematic review service. Poster presented at MLA '13, the 113th Annual Meeting of the Medical Library Association; Boston, MA; May 3–8, 2013.

Menzies E. Use analysis of Scopus for systematic review. J Can Health Libr Assoc. 2015;36(2):85.

Nourbakhsh E, Nugent R, Wang H, Cevik C, Nugent K. Medical literature searches: a comparison of PubMed and Google Scholar. Health Inf Libr J. 2012 Sep;29(3):214–22.

Patel MR, Schardt CM, Sanders LL, Keitz SA. Randomized trial for answers to clinical questions: evaluating a pre-appraised versus a MEDLINE search protocol. J Med Libr Assoc. 2006 Oct;94(4)382–7.

Paynter RA. Cochrane complementary and alternative medicine systematic reviews: an analysis of authors' comments on the quality and quantity of evidence and efficiency conclusions. Presented at MLA '12, the 112th Annual Meeting of the Medical Library Association; Seattle, WA; May 18–23, 2012.

Rethlefsen ML. Can a quality systematic review have a single author? Poster presented at MLA '15, the 115th Annual Meeting of the Medical Library Association; Austin, TX; May 15–20, 2015.

Sampson M, McGowan J. Errors in search strategies were identified by type and frequency. J Clin Epidemiol. 2006 Oct;59(10):1057–63.

Schmidt CM, Cox R, Fial AVV, Hartman TL, Magee ML. Gaps in affiliation indexing in Scopus and PubMed. J Med Libr Assoc. 2016 Apr;104(2):138–42. DOI: http://dx.doi.org/10.3163/1536-5050.104.2.008.

Sheffield CL. Assessing the value of EMBASE. Poster presented at MLA '06, the 106th Annual Meeting of the Medical Library Association; Phoenix, AZ; May 19–24, 2006.

Shultz M. Comparing test searches in PubMed and Google Scholar. J Med Libr Assoc. 2007 Oct;95(4):442–5. DOI: http://dx.doi.org/10.3163/1536-5050.95.4.442.

Topfer LA, Parada A, Menon D, Noorani H, Perras C, Serra-Prat M. Comparison of literature searches on quality and costs for health technology assessment using the MEDLINE and EMBASE databases. Int J Technol Assess Health Care. 1999 Spring;15(2):297–303.

Vincent S. Beyond therapy: evidence based diagnosis—quality of existing systematic reviews and feasibility of searching the literature. Presented at MLA '03, the 103rd Annual Meeting of the Medical Library Association; San Diego, CA; May 2–7, 2003.

Waffenschmidt S, Hausner E, Kaiser T. An evaluation of searching the CCMed database for the production of systematic reviews. Health Inf Libr J. 2010 Dec;27(4):262–7.

Wong SSL, Wilczynski NL, Haynes RB. Comparison of top-performing search strategies for detecting clinically sound treatment studies and systematic reviews in MEDLINE and EMBASE. J Med Libr Assoc. 2006 Oct;94(4):451–5.

<u>Formalized systematic review services</u>

Anderson PF, Ginier EC, Mani NS. Systematic review services on health sciences library websites. Poster presented at MLA '16, the 116th Annual Meeting of the Medical Library Association; Toronto, ON, Canada; May 13–18, 2016.

Farrell A, Brigham T, Marks LA, Golden AK, Osterhaus Trzasko L, Schram J, Almader-Douglas D, Bongiorno CM, Hoy M. Building the big picture: creating a systematic review process across a multiple-site, multi-library system. Poster presented at MLA '16, the 116th Annual Meeting of the Medical Library Association; Toronto, ON, Canada; May 13–18, 2016.

Foster MJ. Developing and evaluating a systematic review service. Poster presented at MLA '13, the 113th Annual Meeting of the Medical Library Association; Boston, MA; May 3–8, 2013.

Fowler SA, Yaeger LH, Kelly B. Creating and managing a systematic review service. Poster presented at MLA ′13, the 113th Annual Meeting of the Medical Library Association; Boston, MA; May 3–8, 2013.

Knehans A, Dell E. Establishing, marketing, and expanding a fee-based systematic review information service. Poster presented at MLA ′15, the 115th Annual Meeting of the Medical Library Association; Austin, TX; May 15–20, 2015.

Knehans A, Dell E, Robinson C. Starting a fee-based systematic review service. Med Ref Serv Q. 2016 Jul–Sep;35(3):266–73.

Krause K, Varman BG, Galati M, Huynh N. Systematic review: the evolution of a new library service. Presented at MLA ′16, the 116th Annual Meeting of the Medical Library Association; Toronto, ON, Canada; May 13–18, 2016.

Ludeman E, Downton K, Fu Y, Goldstein Shipper A. From the ground up: building a successful systematic review service. Poster presented at MLA ′14, the 114th Annual Meeting of the Medical Library Association; Chicago, IL; May 16–21, 2014.

Ludeman E, Downton K, Shipper AG, Fu Y. Developing a library systematic review service: a case study. Med Ref Serv Q. 2015;34(2):173–80.

Qiu KM, Campbell F, Morgan S, Lewis G. Development of a tiered systematic review service model. Poster presented at MLA ′15, the 115th Annual Meeting of the Medical Library Association; Austin, TX; May 15–20, 2015.

Safranek S, St. Anna LA, Dodson S, Schnall JG. Formalizing a systematic review service: librarian collaboration in clinical and scientific research. Poster presented at MLA ′13, the 113th Annual Meeting of the Medical Library Association; Boston, MA; May 3–8, 2013.

Schardt C, Leonardelli A. Systematically supporting research: standardizing our systematic review search service. Poster presented at MLA ′13, the 113th Annual Meeting of the Medical Library Association; Boston, MA; May 3–8, 2013.

Varman BG, Kogut A, Justice A, Krause K, Galati M. Developing a systematic review service in six months. Poster presented at MLA ′15, the 115th Annual Meeting of the Medical Library Association; Austin, TX; May 15–20, 2015.

Impact/outcomes

Allen T. Medical librarians' educational needs in LMIC: systematic review production as an indicator. J Eur Assoc Health Inf Libr. 2016;12(4):17–9.

Desmeules R, Campbell S, Dorgan M. Acknowledging librarians' contributions to systematic review searching. J Can Health Libr Assoc. 2016;37(2):44–52.

Koffel JB. Use of recommended search strategies in systematic reviews and the impact of librarian involvement: a cross-sectional survey of recent authors. PLOS One. 2015 May 4;10(5):e0125931.

Li L, Tian J, Tian H, Moher D, Liang F, Jiang T, Yao L, Yang K. Network meta-analyses could be improved by searching more sources and by involving a librarian. J Clin Epidemiol. 2014 Sep;67(9):1001–7.

Meert D, Torabi N, Costella J. Impact of librarians on reporting of the literature searching component of pediatric systematic reviews. J Med Libr Assoc. 2016 Oct;104(4):267–77. DOI: http://dx.doi.org/10.3163/1536-5050.104.4.004.

JMLA Journal of the Medical Library Association

Supplemental content to
J Med Libr Assoc. Jan;106(1):dx.doi.org/10.5195/jmla.2018.82
www.jmla.mlanet.org
© Spencer, Eldredge, 2018

Medical Library Association. Role of expert searching in health sciences libraries: policy statement by the Medical Library Association adopted September 2003. J Med Libr Assoc. 2005 Jan;93(1):42–4.

Metzendorf MI. Why medical information specialists should routinely form part of teams producing high quality systematic reviews–a Cochrane perspective. J Eur Assoc Health Inf Libr. 2016;12(4):6–9.

Rethlefsen ML, Murad MH, Livingston EH. Engaging medical librarians to improve the quality of review articles. JAMA. 2014 Sep 10;312(1):999–1000.

Rethlefsen ML, Farrell AM, Osterhaus Trzasko LC, Brigham TJ. Librarian co-authors correlated with higher quality reported search strategies in general internal medicine systematic reviews. J Clin Epidemiol. 2015 Jun;68(6):617–26.

Rethlefsen ML, Farrell A, Osterhaus Trzasko LC. Systematic review reporting quality in general medical journals: the influence of librarian authorship. Presented at MLA ′13, the 113th Annual Meeting of the Medical Library Association; Boston, MA; May 3–8, 2013.

Van Noord MG, Ledbetter L, Mazure ES, Tuttle B, von Isenburg M, Carden V. Systematic reviews' impact and the role of the medical librarian. Poster presented at MLA ′15, the 115th Annual Meeting of the Medical Library Association; Austin, TX; May 15–20, 2015.

Weller AC. Mounting evidence that librarians are essential for comprehensive literature searches for meta-analyses and Cochrane reports. J Med Libr Assoc. 2004 Apr;92(2):163–4.

Santesso N. Emphasis on the need for guidelines for documentation of search strategy and results was needed, criticism of a Cochrane review was not [letter to the editor]. J Med Libr Assoc. 2004 Oct;92(4):393–4.

Zhang L, Sampson M, McGowan J. Reporting of the role of the expert searcher in Cochrane Reviews. Evid Based Libr Inf Pract. 2006;1(4):3–16.

Indexing of database terms

Anderson M. Isn't MeSH enough? Medical Subject Headings for systematic review searching: a preliminary look. Poster presented at MLA ′16, the 116th Annual Meeting of the Medical Library Association; Toronto, ON, Canada; May 13–18, 2016.

Funk ME, Reid CA. Indexing consistency in MEDLINE. Bull Med Libr Assoc. 1983 Apr;71(2):176–83.

Wilczynski NL, Haynes RB. Consistency and accuracy of indexing systematic review articles and meta-analysis in MEDLINE. Health Inf Libr J. 2009 Sep;26(3):203–10.

Peer review of search strategies

Crumley E, Bhatnagar N, Stobart K. Peer reviewing comprehensive search strategies in hemophilia and von Willebrand disease. J Can Health Libr Assoc. 2004;25(4):113–6.

McGowan J, Sampson M, Salzwedel DM, Cogo E, Foerster V, Lefebvre C. PRESS Peer Review of Electronic Search Strategies: 2015 guideline statement. J Clin Epidemiol. 2016 Jul;75:40–6.

Paynter R, Relevo R. Peer review of comparative effectiveness review search strategies. Presented at MLA ′12, the 112th Annual Meeting of the Medical Library Association; Seattle, WA; May 18–23, 2012.

Sampson M, McGowan J, Cogo E, Grimshaw J, Moher D, Lefebvre C. An evidence-based practice guideline for the peer review of electronic search strategies. J Clin Epidemiol. 2009 Sep;62(9)944–52.

Sampson M, McGowan JL, Salzwedel DM, Cogo E, Lefebvre C. PRESS: Peer Review of Electronic Search Strategies 2015 updated guideline statement. Presented at MLA ′16, the 116th Annual Meeting of the Medical Library Association; Toronto, ON, Canada; May 13–18, 2016.

Planning

Byrd G. Planning and implementing a systematic review of the literature. Presented at MLA ′04, at the 104th Annual Meeting of the Medical Library Association; Washington, DC; May 21–26, 2004.

Goode V, Lobner K. Setting expectations: getting your systematic review started on the right foot. Poster presented at MLA ′13, the 113th Annual Meeting of the Medical Library Association; Boston, MA; May 3–8, 2013.

Question formulation

Eldredge JD, Carr R, Broudy, D, Voorhees RE. The effect of training on question formulation among public health practitioners: results from a randomized controlled trial. J Med Libr Assoc. 2008 Oct;96(4):299–309. DOI: http://dx.doi.org/10.3163/1536-5050.96.4.005.

Eldredge JD. Evidence-based practice. In: Wood MS, ed. Introduction to health sciences librarianship. New York, NY: Haworth Press; 2008. p. 245–69.

Eldredge JD, Hannigan GG. Emerging trends in health sciences librarianship. In: Health sciences librarianship. Wood MS, ed. Chicago, IL: Rowman & Littlefield; Medical Library Association; 2014. p. 57–83.

Reporting/documenting

Booth A. "Brimful of STARLITE": toward standards for reporting literature searches. J Med Libr Assoc. 2006 Oct;94(4):421–9. e205.

Sandelowski M. In response to "Brimful of STARLITE" [letter to the editor]. J Med Libr Assoc. 2007 Jul;95(3):233. DOI: http://dx.doi.org/10.3163/1536-5050.95.3.233.

Craven J, Levay P. Recording database searches for systematic reviews - what is the value of adding a narrative to peer-review checklists? a case study of NICE interventional procedures guidance. Evid Based Libr Inf Pract. 2011;6(4).

Hutton B, Salanti G, Chaimani A, Caldwell DM, Schmid C, Thorlund K, Mills E, Catalá-Lopez F, Turner L, Altman DG, Moher D. The quality of reporting methods and results in network meta-analyses: an overview of reviews and suggestions for improvement. PLOS One. 2014 Mar;9(3):e92508.

Plaut D, McGraw KA, Anderson MJ, Nguyen L, Wellik KE, Yoshii A. Analysis of the reporting of search strategies in Cochrane systematic reviews. Poster presented at MLA ′07, the 107th Annual Meeting of the Medical Library Association; Philadelphia, PA; May 18–23, 2007.

Rader T, Mann M, Stansfield C, Cooper C, Sampson M. Methods for documenting systematic review searches: a discussion of common issues. Res Synth Methods. 2014 Jun;5(2):98–115.

Rethlefsen M, Farrell A, Osterhaus Trzasko LC. Systematic review reporting quality in general medical journals: the influence of librarian authorship. Presented at MLA ′13, the 113th Annual Meeting of the Medical Library Association; Boston, MA; May 3–8, 2013.

Toews L. Compliance of veterinary medicine systematic reviews with literature search reporting standards. Presented at MLA ′13, the 113th Annual Meeting of the Medical Library Association; Boston, MA; May 3–8, 2013.

Supplemental content to
J Med Libr Assoc. Jan;106(1):dx.doi.org/10.5195/jmla.2018.82
www.jmla.mlanet.org
© Spencer, Eldredge, 2018

Yoshii A, Plaut DA, McGraw KA, Anderson MJ, Wellik KE. Analysis of the reporting of search strategies in Cochrane systematic reviews. J Med Libr Assoc. 2009 Jan;97(1):21–9. DOI: http://dx.doi.org/10.3163/1536-5050.97.1.004.

Research agenda

Ascher MT, Holmes HN, Eldredge JD. Addressing the MLA research agenda questions: where are we now? Poster presented at MLA ′13, the 113th Annual Meeting of the Medical Library Association; Boston, MA; May 3–8, 2013.

Ascher MT, Holmes HN, Eldredge JD. The MLA research agenda: what do we know? the systematic review project: a status report. Poster presented at MLA ′14, the 114th Annual Meeting of the Medical Library Association; Chicago, IL; May 16–21, 2014.

Eldredge JD, Ascher MT, Holmes HN. An innovative model of evidenced-based practice for other professions. J Med Libr Assoc. 2015 Apr;103(2):100–2. DOI: http://dx.doi.org/10.3163/1536-5050.103.2.009.

Eldredge JD, Ascher MT, Holmes HN, Harris MR. Top-ranked research questions and systematic reviews. Hypothesis. 2013;24(2):19–20.

Harris MR, Holmes HN, Ascher MT, Eldredge JD. Inventory of research questions identified by the MLA research agenda delphi study. Hypothesis. 2013;24(2):5–16.

Henderson M, Brown RE, Foster MJ, Klem ML, Ayala AP, Ettien A, McCrillis A, Anderson PF, Swanberg SM, McGowan SS, Castelli D, Heskett K, Linares BM, Woznica A. The MLA research agenda systematic review project. Presented at MLA ′15, the 115th Annual Meeting of the Medical Library Association; Austin, TX; May 15–20, 2015.

Search filters/hedges

Bak G, Mierzwinski-Urban M, Fitzsimmons H, Morrison A, Maden-Jenkins M. A pragmatic critical appraisal instrument for search filters: introducing the CADTH CAI. Health Inf Libr J. 2009 Sep;26(3):211–9.

Beale S, Duffy S, Glanville J, Lefebvre C, Wright D, McCool R, Varley D, Boachie C, Fraser C, Harbour J, Smith L. Choosing and using methodological search filters: searchers' views. Health Inf Libr J. 2014 Jun;31(2):133–47.

Bonato S, Lightfoot D. Developing search hedges for MEDLINE/PsycINFO searches on Aboriginal/Native American people. Presented at MLA ′16, the 116th Annual Meeting of the Medical Library Association; Toronto, ON, Canada; May 13–18, 2016.

Bradley SM. Examination of the clinical queries and systematic review "hedges" in EMBASE and MEDLINE. J Can Health Libr Assoc. 2010;31(2):27–37.

Campbell S, Dorgan M, Tjosvold L. Creating provincial and territorial search filters to retrieve studies related to Canadian Indigenous peoples from Ovid MEDLINE. J Can Health Libr Assoc. 2014;35(1):5–10.

Chatterly T, Dennett L. Utilisation of search filters in systematic reviews of prognosis questions. Health Inf Libr J. 2012 Dec;29(4):309–22.

Clar M, Dupont P. Evidence-based practice in PubMed: are shared search filters useful to health sciences academic users? Poster presented at MLA ′12, the 112th Annual Meeting of the Medical Library Association; Seattle, WA; May 18–23, 2012.

El Sherif R, Pluye P, Gore G, Granikov V, Hong QN. Performance of a mixed filter to identify relevant studies for mixed studies reviews. J Med Libr Assoc. 2016 Jan;104(1):47–51. DOI: http://dx.doi.org/10.3163/1536-5050.104.1.007.

Farrah K, Mierzwinski-Urban M. Playing it safe: validating search filters for adverse events. Poster presented at MLA '13, the 113th Annual Meeting of the Medical Library Association; Boston, MA; May 3–8, 2013.

Farrah K, Mierzwinski-Urban M, Cimon K. Effectiveness of adverse effects search filters: drugs versus medical devices. J Med Libr Assoc. 2016 Jul;104(3):221–5. DOI: http://dx.doi.org/10.3163/1536-5050.104.3.007.

Fowler SA, Yaeger LH, Kelly B, Carpenter CR. Validating a search filter for diagnosis sensitivity and specificity. Poster presented at MLA '13, the 113th Annual Meeting of the Medical Library Association; Boston, MA; May 3–8, 2013.

Frazier JJ, Stein CD, Tseytlin E, Bekhuis T. Building a gold standard to construct search filters: a case study with biomarkers for oral cancer. J Med Libr Assoc. 2015 Jan;103(1)22–30. DOI: http://dx.doi.org/10.3163/1536-5050.103.1.005.

Ganshorn H. Translation of hedges in medical databases to other platforms' syntax may cause significantly different search results. Evid Based Libr Inf Pract. 2011;6(2):55–8.

Glanville J, Arber M, Garcia S, Veale T. Sensitivity of a search filter designed to identify studies reporting health state utility values. Presented at MLA '16, the 116th Annual Meeting of the Medical Library Association; Toronto, ON, Canada; May 13–18, 2016.

Glanville J, Bayliss S, Booth A, Dundar Y, Fernandes H, Fleeman ND, Foster L, Fraser C, Fry-Smith A, Golder S, Lefebvre C, Miller C, Paisley S, Payne L, Price A, Welch K. So many filters, so little time: the development of a search filter appraisal checklist. J Med Libr Assoc. 2008 Oct;96(4)356–61. DOI: http://dx.doi.org/10.3163/1536-5050.96.4.011.

Golder S, Loke YK. Sensitivity and precision of adverse effects search filters in MEDLINE and EMBASE: a case study of fractures with thiazolidinediones. Health Inf Libr J. 2012 Mar;29(1) 28–38.

Golder S, Loke YK. The performance of adverse effects search filters in MEDLINE and EMBASE. Health Inf Libr J. 2012 Jun;29(2):141–51.

Harbour J, Fraser C, Lefebvre C, Glanville J, Beale S, Boachie C, Duffy S, McCool R, Smith L. Reporting methodological search filter performance comparisons: a literature review. Health Inf Libr J. 2014 Sep;31(3):176–94.

Hoogendam A, deVris Robbé PF, Stalenhoef AFH, Overbeke AJPM. Evaluation of PubMed filters used for evidence-based searching: validation using relative recall. J Med Libr Assoc. 2009 Jul;97(3):186–93. DOI: http://dx.doi.org/10.3163/1536-5050.97.3.007.

Jenkins M. Evaluation of methodological search filters--a review. Health Inf Libr J. 2004 Sep;21(3):148–63.

Jenkins M, Johnson F. Awareness, use and opinions of methodological search filters used for the retrieval of evidence-based medical literature-a questionnaire survey. Health Inf Libr J. 2004 Mar;21(1):33–43.

Johnson ED, McKinin EJ, Sievert M. The application of quality filters in searching the clinical literature: some possible heuristics. Med Ref Serv Q. 1992 Winter;11(4):39–59.

Supplemental content to
J Med Libr Assoc. Jan;106(1):dx.doi.org/10.5195/jmla.2018.82
www.jmla.mlanet.org
© Spencer, Eldredge, 2018

Kelly K. Applying the narrow forms of PubMed methods-based and topic-based filters increases nephrologists' search efficiency. Evid Based Libr Inf Pract. 2012;7(3):95–7.

Meadows SE, Hitchcock K, Nashelsky J, Ward DH. Enhancing the systematic search process for quality information retrieval and delivery: filters and a reporting tool for librarian searchers for the Family Physicians Inquires Network (FPIN). Presented at MLA '07, the 107th Annual Meeting of the Medical Library Association; Philadelphia, PA; May 18–23, 2007.

McKibbon KA, Wilczynski NL, Haynes RB; Hedges Team. Retrieving randomized controlled trials from MEDLINE: a comparison of 38 published search filters. Health Inf Libr J. 2009 Sep;26(3):187–202.

Murphy SA. Applying methodological search filters to CAB abstracts to identify research for evidence-based veterinary medicine. J Med Libr Assoc. 2002 Oct;90(4):406–10.

Murphy SA. Research methodology search filters: are they effective for locating research for evidence-based veterinary medicine in PubMed? J Med Libr Assoc. 2003 Oct;91(4):484–9.

Pienaar E, Grobler L, Busgeet K, Eisinga A, Siegfried N. Developing a geographic search filter to identify randomised controlled trials in Africa: finding the optimal balance between sensitivity and precision. Health Inf Libr J. 2011 Sep;28(3):210–5.

Ritchie G, Glanville J, Lefebvre C. Do published search filters to identify diagnostic test accuracy studies perform adequately? Health Inf Libr J. 2007 Sep;24(3):188–92.

Rogers M, Bethel A, Boddy K. Development and testing of a MEDLINE search filter for identifying patient and public involvement in health research. Health Inf Libr J. 2017 Jun;34(2):125–33. DOI: http://dx.doi.org/10.1111/hir.12157.

Sladek R, Tieman J, Fazekas BS, Abernethy AP, Currow DC. Development of a subject search filter to find information relevant to palliative care in the general medical literature. J Med Libr Assoc. 2006 Oct;94(4):394–401.

Skidmore B, Lang R, Abdelfattah D, Wright HL, Seely D, Thiel A, Zhao L, Green J. The development of highly sensitive search filters for complementary and alternative medicine–specific, natural therapies for use in integrative oncology. Presented at MLA '16, the 116th Annual Meeting of the Medical Library Association; Toronto, ON, Canada; May 13–18, 2016.

Skidmore B, Radar T, Daniel RS, Mayhew A, Wieland LS. Creation of complementary and alternative medicine (CAM) search filters for the Cochrane Complementary Medicine Specialized Register of Trials. Poster presented at MLA '16, the 116th Annual Meeting of the Medical Library Association; Toronto, ON, Canada; May 13–18, 2016.

Wilczynski NL, Lokker C, McKibbon KA, Hobson N, Haynes RB. Limits of search filter development. J Med Libr Assoc. 2016 Jan;104(1):42–6. DOI: http://dx.doi.org/10.3163/1536-5050.104.1.006.

Searching

General

Bramer WM, de Jonge GB. Efficiently searching for systematic reviews – how to perform high-quality searches more efficiently. Presented at MLA '15, the 115th Annual Meeting of the Medical Library Association; Austin, TX; May 15–20, 2015.

Fowler SA, Yaeger LH, Hardi AC, Simon LE, Doering M. Systematic reviews: evidence-based searching to improve recall and precision. Presented at MLA '16, the 116th Annual Meeting of the Medical Library Association; Toronto, ON, Canada; May 13–18, 2016.

McGowan J, Sampson M. Systematic reviews need systematic searchers. J Med Libr Assoc. 2005 Jan;93(1):74–80.

Munger H, Brower SM, Buchinger K, Murphy S, Pirrung TE, Wilson S, Lyons AG. Extreme searching: one library's experience with conducting systematic reviews. Presented at MLA '03, the 103rd Annual Meeting of the Medical Library Association; San Diego, CA; May 2–7, 2003.

Riesenberg LA, Justice EM. Conducting a successful systematic review of the literature, part 1. Nursing. 2014 Apr;44(4):13–7.

Sandieson RW, Sandieson RM. Locating systematic reviews comprehensively and efficiently. Presented at MLA '16, the 116th Annual Meeting of the Medical Library Association; Toronto, ON, Canada; May 13–18, 2016.

<u>Database and other resources</u>

Abhijnhan A, Surcheva Z, Wright J, Adams CE. Searching a biomedical bibliographic database from Bulgaria: the ABS database. Health Inf Libr J. 2007 Sep;24(3):200–3.

Almerie MQ, Matar HE, Jones V, Kumar A, Wright J, Wlostowska E, Adams CE. Searching the Polish Medical Bibliography (Polska Bibliografia Lekarska) for trials. Health Inf Libr J. 2007 Dec;24(4):283–6.

Atsawawaranunt K, Adams CE, Roberts S. Searching for randomised controlled trials and clinical controlled trials in Thai online bibliographical biomedical databases. Health Inf Libr J. 2011 Mar;28(1):68–76.

Bachmann LM, Estermann P, Kronenberg C, ter Riet G. Identifying diagnostic accuracy studies in EMBASE. J Med Libr Assoc. 2003 Jul;91(3):341–6.

Chapman AL, Morgan LC, Gartlehner G. Semi-automating the manual literature search for systematic reviews increases efficiency. Health Inf Libr J. 2010 Mar;27(1):22–7.

Glanville JM, Duffy S, McCool R, Varley D. Searching ClinicalTrials.gov and the International Clinical Trials Registry Platform to inform systematic reviews: what are the optimal search approaches? J Med Libr Assoc. 2014 Jul;102(3):177–83. DOI: http://dx.doi.org/10.3163/1536-5050.102.3.007.

Holland JL, Wilczynski NL, Haynes RB; Hedges Team. Optimal search strategies for identifying sound clinical prediction studies in EMBASE. BMC Med Inform Decis Mak. 2005 Apr 29;5:11.

Lefebvre C, Eisinga A, Chapman S. Improving access to reports of randomized controlled trials by searching EMBASE. Poster presented at MLA '09, the 109th Annual Meeting of the Medical Library Association; Honolulu, HI; May 15–20, 2009.

Royle P, Waugh N. Should systematic reviews include searches for published errata? Health Inf Libr J. 2004 Mar;21(1):14–20.

Wong SSL, Wilczynski NL, Haynes RB. Developing optimal search strategies for detecting clinically sound treatment studies in EMBASE. J Med Libr Assoc. 2006 Jan;94(1):41–7.

Gray literature

Ford C, Farrah K, Lefebvre C, Rethlefsen ML, Sampson M. Where does gray fit into the mosaic? a discussion of the use, values, and practicality of gray literature in systematic reviews. Presented at MLA '16, the 116th Annual Meeting of the Medical Library Association; Toronto, ON, Canada; May 13–18, 2016.

Saleh A, Ratajeski MA, Bertolet M. Grey literature searching in health sciences systematic reviews: a prospective study of time spent and resources utilized. Evid Based Libr Inf Pract. 2014;9(3):28–50.

Saleh A, Ratajeski MA, Bertolet M. Grey literature searching in health sciences systematic reviews: a survey study. Poster presented at MLA '12, the 112th Annual Meeting of the Medical Library Association; Seattle, WA; May 18–23, 2012.

Saleh AA, Billman BL. A proposal for a health sciences gray literature resources database. Poster presented at MLA '15, the 115th Annual Meeting of the Medical Library Association; Austin, TX; May 15–20, 2015.

Stapleton J, Godin KM, Kirkpatrick SI, Hanning RM, Leatherdale ST. Systematic search and reporting techniques applied to the gray literature: a review of Canadian school breakfast program guidelines. Poster presented at MLA '16, the 116th Annual Meeting of the Medical Library Association; Toronto, ON, Canada; May 13–18, 2016.

Protocol development

Agha R, Fowler AJ, Lee SY, Gundogan B, Whitehurst K, Sagoo H, Jeong K, Altman DG, Orgill DP. A systematic review protocol for reporting deficiencies within surgical case series. BMJ Open. 2015 Oct 5;5(10):e008007.

Arigoni S, Ignjatovic S, Sager P, Betschart J, Buerge T, Wachtl J, Tschuor C, Limani P, Puhan MA, Lesurtel M, Raptis DA, Breitenstein S. Diagnosis and prediction of neuroendocrine liver metastases: a protocol of six systematic reviews. JMIR Res Protoc. 2013 Dec 23;23(2):e60.

Limani,P, Tschuor C, Gort L, Balmer B, Gu A, Ceresa C, Raptis DA, Lesurtel M, Puhan M, Breitenstein S. Nonsurgical strategies in patients with NET liver metastases: a protocol of four systematic reviews. JMIR Res Protoc. 2014 Mar 7;3(1):e9.

McCool ME, Theurich MA, Apfelbacher C. Prevalence and predictors of female sexual dysfunction: a protocol for a systematic review. Syst Rev. 2014 Jul 11;3:75.

Search strategies

Crumley E, Blackhall K. Setting up search strategies for systematic reviews (or, how many ways can you spell diarrhea?). Bibl Med Can. 2003;24(4):167–8.

DeLuca JB, Mullins MM, Lyles CM, Crepaz N, Kay L, Thadiparthi S. Developing a comprehensive search strategy for evidence based systematic reviews. Evid Based Libr Inf Pract. 2008;3(1):3–32.

Grant MJ. How does your searching grow? a survey of search preferences and the use of optimal search strategies in the identification of qualitative research. Health Inf Libr J. 2004;21(1):21–32.

Subject or topic specific searches

Alborz A, McNally R. Developing methods for systematic reviewing in health services delivery and organization: an example from a review of access to health care for people with learning disabilities. part 2. evaluation of the literature--a practical guide. Health Inf Libr J. 2004 Dec;21(4):227–36.

Arendt J. How do psychology researchers find studies to include in meta-analyses? Behav Soc Sci Libr. 2007;26(1):1–23.

Beahler CC, Sundheim JJ, Trapp NI. Information retrieval in systematic reviews: challenges in the public health arena. Am J Prev Med. 2000 May;18(4 suppl):6–10.

Beynon R, Leeflang MM, McDonald S, Eisinga A, Mitchell RL, Whiting P, Glanville JM. Search strategies to identify diagnostic accuracy in MEDLINE. Cochrane Database Syst Rev. 2013 Sep 11;(9):MR000022.

Booth A, Carroll C. Systematic searching for theory to inform systematic reviews: is it feasible? is it desirable? Health Inf Libr J. 2015 Sep;32(3):220–35.

de Kock S, Misso K, Stirk L, Westwood M, Deshpande S, Kleijnen J, Clayton D, Kleijnen J. Finding systematic reviews on pain: building the KSR Pain Evidence database. J Eur Assoc Health Inf Libr. 2016;12(3):18–20.

de Kock S, Westwood M, Misso K, Stirk L, Deshpande S, Kleijnen J, McLellan A, Kleijnen J. Developing a database of critically appraised systematic reviews in the field of pain management. Poster presented at MLA '16, the 116th Annual Meeting of the Medical Library Association; Toronto, ON, Canada; May 13–18, 2016.

Golder S, McIntosh HM, Duffy S, Glanville J; Centre for Reviews and Dissemination and UK Cochrane Centre Search Filters Design Group. Developing efficient search strategies to identify reports of adverse effects in MEDLINE and EMBASE. Health Inf Libr J. 2006 Mar;23(1):3–12.

Golder S. Optimising the retrieval of information on adverse drug effects. Health Inf Libr J. 2013 Dec;30(4):327–31.

Golder S, Loke Y. Search strategies to identify information on adverse effects: a systematic review. J Med Libr Assoc. 2009 Apr;97(2):84–92. DOI: http://dx.doi.org/10.3163/1536-5050.97.2.004.

Golder S, Mason A, Spilsbury K. Systematic searches for the effectiveness of respite care. J Med Libr Assoc. 2008 Apr;96(2):147–52. DOI: http://dx.doi.org/10.3163/1536-5050.96.2.147.

Papaioannou D, Sutton A, Carrol, C, Booth A, Wong R. Literature searching for social science systematic reviews: consideration of a range of search techniques. Health Inf Libr J. 2010 Jun;27(2):114–22.

Parker R, Tougas R, Hayden J. When the RCT filter is not enough: best practices for finding prognosis studies. J Can Health Libr Assoc. 2014;35(2):99–100.

Vassar M, Atakpo P, Kash MJ. Manual search approaches used by systematic reviewers in dermatology. J Med Libr Assoc. 2016 Oct;104(4):302–4. DOI: http://dx.doi.org/10.3163/1536-5050.104.4.009.

Vincent S, Greenley S, Beaven O. Clinical Evidence diagnosis: developing a sensitive search strategy to retrieve diagnostic studies on deep vein thrombosis: a pragmatic approach. Health Inf Libr J. 2003 Sep;20(3):150–9.

Walsh ES, Peterson JJ, Judkins DZ; Expert Panel on Health Care Disparities Among Individuals with Disabilities. Searching for disability in electronic databases of published literature. Disabil Health J. 2014 Jan;7(1):114–8.

Woodman J, Harden A, Thomas J, Brunton J, Kavanagh J, Stansfield C. Searching for systematic reviews of the effects of social and environmental interventions: a case study of children and obesity. J Med Libr Assoc. 2010 Apr;98(2):140–6. DOI: http://dx.doi.org/10.3163/1536-5050.98.2.006.

Supplemental content to
J Med Libr Assoc. Jan;106(1):dx.doi.org/10.5195/jmla.2018.82
www.jmla.mlanet.org
© Spencer, Eldredge, 2018

Other

Bramer WM. Optimizing of s7 ystematic review searches: when can you stop? Poster presented at MLA ʼ16, the 116th Annual Meeting of the Medical Library Association; Toronto, ON, Canada; May 13–18, 2016.

Duffy S, de Kock S, Misso K, Noake C, Ross J, Stirk L. Supplementary searches of PubMed to improve currency of MEDLINE and MEDLINE In-Process searches via Ovid. J Med Libr Assoc. 2016 Oct;104(4):309–12. DOI: http://dx.doi.org/10.3163/1536-5050.104.4.011.

Glanville JM, Lefebvre C, Miles JNV, Camosso-Stefinovic J. How to identify randomized controlled trials in MEDLINE: ten years on. J Med Libr Assoc. 2006 Apr;94(2):130–6. Correction in: J Med Libr Assoc. 2006 Jul;94(3):354.

Glanville JM, Dooley G, Noel-Storr A, Foxlee R. Improving access to reports of randomized controlled trials in EMBASE: innovative methods enhance the Cochrane Central Register of Controlled Trials (CENTRAL). Presented at MLA ʼ16, the 116th Annual Meeting of the Medical Library Association; Toronto, ON, Canada; May 13–18, 2016.

Jenuwine ES, Floyd JA. Comparison of Medical Subject Headings and text-word searches in MEDLINE to retrieve studies on sleep in healthy individuals. J Med Libr Assoc. 2004 Jul;92(3):349–53.

Lefebvre C, Glanville J, Wieland LS, Coles B, Weightman AL. Methodological developments in searching for studies for systematic reviews: past, present and future? Syst Rev. 2013 Sep 25;2:78.

Posey R, Walker J, Crowell KE. Knowing when to stop: final results versus work involved in systematic review database searching. Presented at MLA ʼ16, the 116th Annual Meeting of the Medical Library Association; Toronto, ON, Canada; May 13–18, 2016.

Relevo R. Using analytic framework to make sense of complex search requests. Poster presented at MLA ʼ10, the 110th Annual Meeting of the Medical Library Association; Washington, DC; May 21–26, 2010.

Source selection

Bethal A, Rogers M. A checklist to assess database-hosting platforms for designing and running searches for systematic reviews. Health Inf Libr J. 2014 Mar;31(1):43–53.

Beyer FR, Wright K. Can we prioritise which databases to search? a case study using a systematic review of frozen shoulder management. Health Inf Libr J. 2013 Mar;30(1):49–58.

Boehm K, Raak C, Vollmar, HC, Ostermann T. An overview of 45 published database resources for complementary and alternative medicine. Health Inf Libr J. 2010 Jun;27(2):93–105.

Bramer WM. The added value of multiple databases in searching for exhaustiveness: a prospective study. Presented at MLA ʼ16, the 116th Annual Meeting of the Medical Library Association; Toronto, ON, Canada; May 13–18, 2016.

Brettle AJ, Long AF. Comparison of bibliographic databases for information on the rehabilitation of people with severe mental illness. Bull Med Libr Assoc. 2001 Oct;89(4):353–62.

Crumley ET, Wiebe N, Cramer K, Klassen TP, Hartling L. Which resources should be used to identify RCT/CCTs for systematic reviews: a systematic review. BMC Med Res Methodol. 2005 Aug 10;5:24.

Glanville JM, Wood H, Arber M. What information resources are searched to prepare systematic reviews of economic evaluations in health care? Presented at MLA ʼ16, the 116th Annual Meeting of the Medical Library Association; Toronto, ON, Canada; May 13–18, 2016.

Golder S, Loke YK. Sources of information on adverse effects: a systematic review. Health Inf Libr J. 2010 Sep;27(3);176–90.

Greenley SL. Connecting with international evidence: measuring the importance of multiple database searching for BMJ Clinical Evidence. Poster presented at MLA ′08, the 108th Annual Meeting of the Medical Library Association; Chicago, IL; May 16–21, 2008.

Greyson DL. Non-biomedical sources for systematic reviews of pharmaceutical policy. J Med Libr Assoc. 2010 Jan;98(1):85–7. DOI: http://dx.doi.org/10.3163/1536-5050.98.1.021.

Helmer D, Savoie I, Green C, Kazanjian A. Evidence-based practice: extending the search to find material for the systematic review. Bull Med Libr Assoc. 2001 Oct;89(4):346–52.

Krevit L. Beyond MEDLINE: exploring resources in evidence-based dentistry. Presented at MLA ′02, the 102nd Annual Meeting of the Medical Library Association; Dallas, TX; May 17–23, 2002.

Lam MT, McDiarmid M. Increasing number of databases searched in systematic reviews and meta-analyses between 1994 and 2014. J Med Libr Assoc. 2016 Oct;104(4):284–9. DOI: http://dx.doi.org/10.3163/1536-5050.104.4.006.

McNally R, Alborz A. Developing methods for systematic reviewing in health services delivery and organization: an example from a review of access to health care for people with learning disabilities. part 1. identifying the literature. Health Inf Libr J. 2004 Sep;21(3):182–92.

Ross-White A, Godfrey CM. Number needed to read: what is the value of searching different databases in systematic reviews? Presented at MLA ′15, the 115th Annual Meeting of the Medical Library Association; Austin, TX; May 15–20, 2015.

Sakmar K, Bullers K, Howard AM, Orriola JJ, Polo RL. Have researchers expanded their limits? resources used in systematic reviews over time. Poster presented at MLA ′15, the 115th Annual Meeting of the Medical Library Association; Austin, TX; May 15–20, 2015.

Sampson M, Daniel R, Cogo E, Dingwall O. Sources of evidence to support systematic reviews in librarianship. J Med Libr Assoc. 2008 Jan;96(1):66–9. DOI: http://dx.doi.org/10.3163/1536-5050.96.1.66.

Vassar M, Carr B, Kash-Holley M, DeWitt E, Koller C, Day J, Day K, Hermann D, Holzmann M. Database choices in endocrine systematic reviews. J Med Libr Assoc. 2015 Oct;103(4):189–92. DOI: http://dx.doi.org/10.3163/1536-5050.103.4.005.

Systematic reviews on medical librarianship

Ascher MT, Crooke DA, Cunningham DJ. Seizing the power of the systematic review for the development of a health literacy curriculum. Presented at MLA ′10, at the 110th Annual Meeting of the Medical Library Association; Washington, DC; May 21–26, 2010.

Boden C, Adamczyk A, Ambriz L, Billman BL, Booth A, Clark E, Engwall K, Johnson R, Miller-Nesbitt A, Morris M, Woznica A. Librarian knowledge and skills of tools for visualizing, mining, and managing large and complex research data: a systematic review. Poster presented at MLA ′15, the 115th Annual Meeting of the Medical Library Association; Austin, TX; May 15–20, 2015.

Booth A, Carroll C, Papaioannou D, Sutton A, Wong R. Applying findings from a systematic review of workplace-based e-learning: implications for health information professionals. Health Inf Libr J. 2009 Mar;26(1):4–21.

Boruff J, Harrison P. Assessment of knowledge and skills in allied health student information literacy instruction: a systematic review. J Can Health Libr Assoc. 2015;36(2):75.

Brettle A, Maden-Jenkins M, Anderson L, McNally R, Pratchett T, Tancock J, Thornton D, Webb A. Evaluating clinical librarian services: a systematic review. Health Inf Libr J. 2011 Mar;28(1):3–22.

Brettle A. Evaluating information skills training in health libraries: a systematic review. Health Inf Libr J. 2007 Dec;24(suppl 1):18–37.

Brettle A. Information skills training: a systematic review of the literature. Health Inf Libr J. 2003 Jun;20(suppl 1):3–9.

Brettle A, Maden M, Payne C. The impact of clinical librarian services on patients and health care organisations. Health Inf Libr J. 2016 Jun;33(2):100–20.

Byrd GD, Wagner KC. Evaluating the effectiveness of clinical medical librarian programs: a systematic review of the literature. Presented at MLA '03, the 103rd Annual Meeting of the Medical Library Association; San Diego, CA; May 2–7, 2003.

Childs S, Blenkinsopp E, Hall A, Walton G. Effective e-learning for health professionals and students--barriers and their solutions. a systematic review of the literature--findings from the HeXL project. Health Inf Libr J. 2005 Dec;22(suppl 2):20–32.

Cooper ID, Crum JA. New activities and changing roles of health sciences librarians: a systematic review, 1990–2012. J Med Libr Assoc. 2013 Oct;101(4):268–77. DOI: http://dx.doi.org/10.3163/1536-5050.101.4.008.

Elueze IN. Evaluating the effectiveness of knowledge brokering in health research: a systematised review with some bibliometric information. Health Inf Libr J. 2015 Sep;32(3):168–81.

Fanner D, Urquhart C. Bibliotherapy for mental health service users part 1: a systematic review. Health Inf Libr J. 2008 Dec;25(4):237–52.

Glynn LA, Berry R, Clemans-Taylor L, Ettien A, Gadd K, Geldenhuys PR, Kim C, Lalla NJ, Lawton A, McClurg C, Sakmar K, Sieber JL. The value to and impact of health sciences libraries and information services on academic and clinical practices: a systematic review. Presented at MLA '14, the 114th Annual Meeting of the Medical Library Association; Chicago, IL; May 16–21, 2014.

Grant MJ. The role of reflection in the library and information sector: a systematic review. Health Inf Libr J. 2007 Sep;24(3):155–66.

Gray H, Sutton G, Treadway V. Do quality improvement systems improve health library services? a systematic review. Health Inf Libr J. 2012 Sep;29(3):180–96.

Henderson M, Crum JA, Fatkin KJ, Gagnon MM, Nguyen T, Taylor M, Vrabel M. Do health sciences libraries and librarians have an impact on the cost of health care and research? a systematic review. Poster presented at MLA '15, the 115th Annual Meeting of the Medical Library Association; Austin, TX; May 15–20, 2015.

Holyoke A, Farrell A, O'Brien K, Dennison CC, Marton C, Thuna M, Kysh L, Swanberg S, Gore G, Pannabecker V. Effectiveness of instructional methods used by librarians for teaching evidence-based practice: a systematic review. J Can Health Libr Assoc. 2014;35(2):92–3.

Madden A, Collins P, McGowan S, Stevenson P, Castelli D, Hyde L, DeSanto K, O'Brien N, Purdon M, Delgado D. Demonstrating the financial impact of clinical libraries: a systematic review. Health Inf Libr J. 2016 Sep;33(3):172–89.

Mairs K, McNeil H, McLeod J, Prorok JC, Stolee P. Online strategies to facilitate health-related knowledge transfer: a systematic search and review. Health Inf Libr J. 2013 Dec;30(4):261–77.

Mi M, Wu W, Qiu KM, Zhang Y, Wu L, Li J. Use of mobile devices to access information resources among health professions students: a systematic review. Poster presented at MLA ’15, the 115th Annual Meeting of the Medical Library Association; Austin, TX; May 15–20, 2015.

Perrier L, Farrell A, Ayala AP, Lightfoot D, Kenny T, Aaronson E, Allee N, Brigham T, Connor E, Constantinescu T, Muellenbach J, Epstein HA, Weiss A. Effects of librarian-provided services in healthcare settings: a systematic review. J Am Med Inform Assoc. 2014 Nov–Dec;21(6):1118–24.

Rankin JA, Grefsheim SF, Canto CC. The emerging informationist specialty: a systematic review of the literature. J Med Libr Assoc. 2008 Jul;96(3):194–206. DOI: http://dx.doi.org/10.3163/1536-5050.96.3.005.

Swanberg SM, Dennison CC, Farrell A, Machel V, Marton C, O’Brien KK, Pannabecker V, Thuna M, Holyoke AN. Instructional methods used by health sciences librarians to teach evidence-based practice (EBP): a systematic review. J Med Libr Assoc. 2016 Jul;104(3):197–208. DOI: http://dx.doi.org/10.3163/1536-5050.104.3.004.

Wagner KC, Byrd GD. Evaluating the effectiveness of clinical medical librarian programs: a systematic review of the literature. J Med Libr Assoc. 2004 Jan;92(1):14–33.

Wagner KC, Byrd GD. Evaluating the effectiveness of clinical medical librarian programs: a systematic review of the literature [reprint]. J Med Libr Assoc. 2012 Oct;100(4 suppl):J.

Weightman AL, Williamson J; Library & Knowledge Development Network (LKDN) Quality and Statistics Group. The value and impact of information provided through library services for patient care: a systematic review. Health Inf Libr J. 2005 Mar;22(1):4–25.

Winning MA, Beverley CA. Clinical librarianship: a systematic review of the literature. Health Inf Libr J. 2003 Jun;20(suppl 1):10–21.

Teaching

Ankem K. Systematic reviews and meta-analysis in health sciences information research: an appraisal and a tutorial. Presented at MLA ’06, the 106th Annual Meeting of the Medical Library Association; Phoenix, AZ; May 19–24, 2006.

Blackstock M. Systematic review training for library users at King’s College London: the past, present and future. ALISS Q. 2015;10(3):13–6.

Campbell SM, Kung JYC, Dennett L. A curriculum for an introductory systematic review searching workshop for researchers. J Can Health Libr Assoc. 2016;37(1):2–5.

Conte ML, Kelley C, MacEachern MP, Mani NS, Smith J. Flipping the classroom: developing and piloting a successful systematic review course for librarians utilizing online and in person instruction. Presented at MLA ’14, the 114th Annual Meeting of the Medical Library Association; Chicago, IL; May 16–21, 2014.

Conte ML, MacEachern, MP, Mani NS, Townsend WA, Smith JE, Masters C, Kelley C. Flipping the classroom to teach systematic reviews: the development of a continuing education course for librarians. J Med Libr Assoc. 2015 Apr;103(2):69–73. DOI: http://dx.doi.org/10.3163/1536-5050.103.2.002.

Fyfe T, Dennett L. Building capacity in systematic review searching: a pilot program using virtual mentoring. J Can Health Libr Assoc. 2012;33(1):12–6.

Harris M. The librarian's role in conducting a systematic review. Presented at MLA 2000, the 100th Annual Meeting of the Medical Library Association; Vancouver, BC, Canada; May 5–11, 2000.

Hartman LM, Folb B, Klem ML, Ratajeski MA, Saleh A, Wessel CB, Ketchum AM. What happens after: outcomes of a systematic review course. Presented at MLA '13, the 113th Annual Meeting of the Medical Library Association; Boston, MA; May 3–8, 2013.

Johnson RE, Kysh L. Blinded ambition: misperceptions and misconceptions about systematic reviews from teachers to learners. Presented at MLA '16, the 116th Annual Meeting of the Medical Library Association; Toronto, ON, Canada; May 13–18, 2016.

Kaplan GE, Benevides TW, Hunter P, Kipnis DG. Supporting the occupational therapy student in the production and dissemination of systematic reviews: an interprofessional collaboration among librarians and occupational therapy faculty. Poster presented at MLA '15, the 115th Annual Meeting of the Medical Library Association; Austin, TX; May 15–20, 2015.

Kopp-Helm D, Wyssmann, BM. Job shadowing - Swiss health librarians observing experienced search specialists and information skills trainers in London. J Eur Assoc Health Inf Libr. 2016;12(1):10–3.

MacEachern MP, Townsend W. Collaborative approach to systematic review and meta-analysis instruction. Presented at MLA '13, the 113th Annual Meeting of the Medical Library Association; Boston, MA; May 3–8, 2013.

Morris M, Boden C, Miller-Nesbitt A. Distributed collaborate virtual systematic reviewing: a blueprint for the future? J Can Health Libr Assoc. 2014;35(2):111–2.

Parker RMN, Neilson MJ. Lost in translation: supporting learners to search comprehensively across databases. J Can Health Libr Assoc. 2015;36(2):54–8.

Parker RMN, Neilson M. Lost in translation: evaluation of support tools for systematic literature search training. J Can Health Libr Assoc. 2014;35(2):80–1.

Parker RMN, Visintini SM, Boulos LMN, Ritchie K, Hayden JA. Supporting knowledge synthesis methods training review of the evidence for online systematic review instruction. Presented at MLA '16, the 116th Annual Meeting of the Medical Library Association; Toronto, ON, Canada; May 13–18, 2016.

Posey R, Walker J, Linares B. Systematic review classes for non-librarians. Poster presented at MLA '16, the 116th Annual Meeting of the Medical Library Association; Toronto, ON, Canada; May 13–18, 2016.

Sieving P, Ryan M, Pilch SM, Smith K, Terry NL, White-Olson A. Development of a class for researchers on best practices for conducting and reporting systematic reviews. Presented at MLA '13, the 113th Annual Meeting of the Medical Library Association; Boston, MA; May 3–8, 2013.

Twose C, Roseman L, Gross P, Hesson DD, Adamo JM, Li T, Saldanaha I, Vedula SS, Dickersin K. An interdisciplinary collaboration to teach systematic review methods. Presented at MLA '13, the 113th Annual Meeting of the Medical Library Association; Boston, MA; May 3–8, 2013.

Tools

Albert P, Delgado D, Piazza A, Richardson JE. Evaluating the usability of systematic review software tools. Presented at MLA '14, the 114th Annual Meeting of the Medical Library Association; Chicago, IL; May 16–21, 2014.

Bekhuis T, Dziabiak MP. Google Translate: a useful tool for librarians and systematic reviews. MLA News. 2011 Nov/Dec;51(10):39.

Brennan D. Conversion of journal citation data for systematic review analysis. Poster presented at MLA '15, the 115th Annual Meeting of the Medical Library Association; Austin, TX; May 15–20, 2015.

Dobbins M, DeCorby K, Ciliska D, Thomas H. An innovative resource to promote evidence-based public health decision making. Presented at MLA '06, the 106th Annual Meeting of the Medical Library Association; Phoenix, AZ; May 19–24, 2006.

Gall C, Brahmi FA. Retrieval comparison of EndNote to search MEDLINE (Ovid and PubMed) versus searching them directly. Med Ref Serv Q. 2004 Fall;23(30):25–32.

Marshall C, Glanville J, McCool R. The systematic review toolbox: finding software to support the systematic review process. J Eur Assoc Health Inf Libr. 2016;12(3):58–9.

Matwin S, Kouznetsov A, Inkpen D, Frunza O, O'Blenis P. A new algorithm for reducing the workload of experts in performing systematic reviews. J Am Med Inform Assoc. 2010 Jul–Aug;17(4):446–53.

McKibbon A, Goldsmith C, Hannigan GG. Is my search complete? the capture mark recapture method (CMR) to estimate the number of citations that are missing. Presented at MLA '10, the 110th Annual Meeting of the Medical Library Association; Washington, DC; May 21–26, 2010.

Nash-Stewart CE, Kruesi LM, Del Mar CB. Does Bradford's Law of Scattering predict the size of the literature in Cochrane reviews? J Med Libr Assoc. 2012 Apr;100(2):135–8. DOI: http://dx.doi.org/10.3163/1536-5050.100.2.013.

Saleh A, Ratajeski M, LaDue J. Development of a collaborative web-based terminology database, a resource for comprehensive search preparation. Presented at MLA '11, the 111th Annual Meeting of the Medical Library Association; Minneapolis, MN; May 13–18, 2011.

Saleh AA, Ratajeski MA, Ladue J. Development of a web-based repository for sharing biomedical terminology form systematic review searches: a case study. Med Ref Serv Q. 2014;33(2):167–78.

Sampson M, McGowan J, Cogo E, Horsley T. Managing database overlap in systematic reviews using Batch Citation Matcher: case studies using Scopus. J Med Libr Assoc. 2006 Oct;94(4):461–3, e219.

Smalheiser NR. A pipeline of informatics tools to accelerate the writing of systematic reviews. Presented at MLA '14, the 114th Annual Meeting of the Medical Library Association; Chicago, IL; May 16–21, 2014.

Stansfield C, O'Mara-Eves A, Thomas J. Reducing systematic review workload using text mining: opportunities and pitfalls. J Eur Assoc Health Inf Libr. 2015;11(3):8–10.

Tsay MY, Yang YH. Bibliometric analysis of the literature of randomized controlled trials. J Med Libr Assoc. 2005 Oct;93(4):450–8.

Witteman W, Englesakis MF, Witteman H, Krahn M. Development of an open source tool to support literature screening for systematic reviews. Presented at MLA '10, the 110th Annual Meeting of the Medical Library Association; Washington, DC; May 21–26, 2010.

Other

Bekhuis T, Tseytlin E, Faith A, Linkov F. A biomedical taxonomy of study designs and publication types: a resource for information professionals who support systematic review teams. Presented at MLA '16, the 116th Annual Meeting of the Medical Library Association; Toronto, ON, Canada; May 13–18, 2016.

Billman BL, Saleh AA. A survey of challenges, barriers, and outcomes of information professionals' involvement in systematic reviews. Poster presented at MLA '15, the 115th Annual Meeting of the Medical Library Association; Austin, TX; May 15–20, 2015.

Supplemental content to
J Med Libr Assoc. Jan;106(1):dx.doi.org/10.5195/jmla.2018.82
www.jmla.mlanet.org
© Spencer, Eldredge, 2018

Boden C, Hellsten L. Evaluation of systematic review knowledge and training needs: supporting systematic review research capacity development. Presented at MLA ′16, the 116th Annual Meeting of the Medical Library Association; Toronto, ON, Canada; May 13–18, 2016.

Bullers K, Howard AM, Sakmar K, Polo RL, Orriola JJ. How long does it take to paint your part of the big picture: the time librarians spend on systematic review tasks. Poster presented at MLA ′16, the 116th Annual Meeting of the Medical Library Association; Toronto, ON, Canada; May 13–18, 2016.

Crum JA, Cooper D. Emerging roles for biomedical librarians: a survey of current practice, challenges, and changes. J Med Libr Assoc. 2013 Oct;101(4):278–86. DOI: http://dx.doi.org/10.3163/1536-5050.101.4.009.

Deberg J, Kiscaden E. Discovering trends in locally published systematic reviews. Poster presented at MLA ′15, the 115th Annual Meeting of the Medical Library Association; Austin, TX; May 15–20, 2015.

de Jonge G, Lein RK. Sharing literature search blocks: status and ideas for a cooperative solution. J Eur Assoc Health Inf Libr. 2015;11(3):11–4.

Heimlich SL. New and emerging roles for medical librarians. J Hosp Librariansh. 2014;14(1):24–32.

Foster MJ. The development of the Systematic Review Special Interest Group of MLA. Poster presented at MLA ′15, the 115th Annual Meeting of the Medical Library Association; Austin, TX; May 15–20, 2015.

Foster MJ, Moberly HK, Halling TD. Development of a value-added database of evaluated systematic reviews in veterinary medicine: DVM Evidence. Presented at MLA ′15, the 115th Annual Meeting of the Medical Library Association; Austin, TX; May 15–20, 2015.

Gore GC, Jones J. Systematic reviews and librarians: a primer for managers. Partnership: Can J Libr Inf Pract Res. 2015;10(1):1–16.

Grant MJ, Booth A. A typology of reviews: an analysis of 14 review types and associated methodologies. Health Inf Libr J. 2009 Jun;26(2):91–108.

Hartley J. Clarifying the abstracts of systematic literature reviews. Bull Med Libr Assoc. 2000 Oct;88(4)332–7.

Murphy SA, Boden C. Benchmarking participation of Canadian university health sciences librarians in systematic reviews. J Med Libr Assoc. 2015 Apr;103(2):73–8. DOI: http://dx.doi.org/10.3163/1536-5050.103.2.003.

Saan MC, Boeije HR, Sattoe JN, Bal MI, Missler M, van Wesel F. Recording and accounting for stakeholder involvement in systematic reviews. Health Inf Libr J. 2015 Jun;32(3):95–106.

Sampson M. Welcoming systematic reviews to the Journal of the Medical Library Association [editorial]. J Med Libr Assoc. 2014 Jul;102(3):143–5. DOI: http://dx.doi.org/10.3163/1536-5050.102.3.001.

Sieving P, Dickersin K, Scherer R, Ervin AM. Proposal for certification of librarians as partners in systematic reviews. Poster presented at MLA ′13, the 113th Annual Meeting of the Medical Library Association; Boston, MA; May 3–8, 2013.

Smith JT Jr, Smith MC, Stullenbarger E. Decision points in the integrative research review process: a flow-chart approach. Med Ref Serv Q. 1991 Summer;10(2):47–72.

Stapleton J, Gordon S, Davies M, Hutchinson R. The evolving role of liaison librarians–supporting researchers in the systematic review process. J Can Health Libr Assoc. 2014;35(2):96.
